# Disparities in Post-Acute Rehabilitation Care for Stroke

**DOI:** 10.1016/j.apmr.2011.03.019

**Published:** 2011-08

**Authors:** Janet K. Freburger, George M. Holmes, Li-Jung Ku, Malcolm Cutchin, Kendra Heatwole-Shank, Lloyd Edwards

**Affiliations:** Institute on Aging; Cecil G. Sheps Center for Health Services Research, University of North Carolina, Chapel Hill, NC; Dept. of Health Policy & Management; Cecil G. Sheps Center for Health Services Research, University of North Carolina, Chapel Hill, NC; Department of Health Policy & Management; Institute on Aging, University of North Carolina, Chapel Hill, NC; Division of Occupational Science; Institute on Aging, University of North Carolina, Chapel Hill, NC; Division of Occupational Science; Institute on Aging, University of North Carolina, Chapel Hill, NC; Department of Biostatistics; Institute on Aging, University of North Carolina, Chapel Hill, NC

**Keywords:** stroke, healthcare disparities, rehabilitation

## Abstract

**Objective:**

To use population-based, hospital discharge data to determine the extent to which demographic and geographic disparities exist in the use of PARC following stroke.

**Design:**

Cross-sectional analysis of two years (2005-2006) of population-based, hospital discharge data.

**Setting:**

All short-term acute care hospitals in four demographically and geographically diverse states (AZ, FL, NJ, WI).

**Participants:**

Individuals 45 years and older (mean age of 72.6 years) admitted to the hospital with a primary diagnosis of stroke, who survived their inpatient stay and who were not transferred to a hospice or other short-term, acute care facility (N=187,188). The sample was 52.4 percent female, 79.5 percent White, 11.4 percent Black, and 9.1 percent Hispanic.

**Interventions:**

Not applicable.

**Main Outcome Measures:**

1) Discharge to an institution versus home. 2) For those discharged home, discharge home with or without home health (HH). 3) For those discharged to an institution, discharge to an inpatient rehabilitation facility (IRF) or skilled nursing facility (SNF). Multilevel logistic regression analyses were conducted to identify demographic and geographic disparities in PARC use, controlling for illness severity/comorbidities, hospital characteristics, and PARC supply.

**Results:**

Blacks, females, older individuals, and those with lower incomes were more likely to be discharged to an institution; Hispanic individuals and the uninsured were less likely. Racial minorities, females, older individuals, and those with lower incomes were more likely to receive HH; uninsured individuals and rural residents were less likely. Blacks, females, older individuals, the uninsured, and those with lower incomes were more likely to use SNF vs IRF care. PARC use varied significantly by state and by hospital.

**Conclusions:**

Several demographic and geographic disparities in PARC use were identified.

## INTRODUCTION

Stroke is a leading cause of death and serious, long-term disability with direct and indirect costs in the US estimated at $73.7 billion for 2010.^1,2^ Each year, approximately 795,000 people experience a new or recurrent stroke, with incident and mortality rates higher in older adults, Blacks, and Hispanics.^1^ Older adults, women, Blacks, and Hispanic stroke survivors also suffer greater morbidity and disability.^3-15^ In addition, research suggests that females, minorities, and individuals of lower socioeconomic status (SES) make smaller gains during post-acute rehabilitation care (PARC) and/or in recovery post-stroke.^4,11,16-19^

A growing body of literature is exploring how demographic and geographic disparities in stroke-related health may be related to demographic and geographic disparities in stroke care. Several studies have documented lower use of thrombolytic treatments for acute stroke ^20-25^ and of preventive measures ^26-30^ in minorities, females, and individuals living in rural areas. Less is known about disparities in rehabilitation care following stroke, an intervention that has a large evidence base for effectiveness.^31-38^ While current guidelines recommend that stroke rehabilitation begin in the acute care setting,^24,39-42^ most of the rehabilitation occurs in post-acute care settings (i.e., skilled nursing facility (SNF), inpatient rehabilitation facility (IRF), home health (HH), and/or outpatient settings).

Reports on the presence of racial disparities in the use of PARC for stroke are mixed and difficult to synthesize due to differences in study context, samples, and measures of PARC. Bhandari et al.^17^ analyzed data from one IRF and reported no racial differences in the intensity of rehabilitative services received by patients with stroke. Horner and colleagues, in an analysis of VA data, reported no racial/ethnic differences in the use of inpatient rehabilitation services following stroke, although low-income Blacks were more likely to experience a delay in the initiation of rehabilitation.^18^ Gregory and colleagues analyzed hospital discharge data from two states and reported race was not associated with IRF use.^43,44^ Urban-dwelling Blacks and Blacks who suffered a hemorrhagic stroke, however, were more likely to receive IRF care in Maryland while individuals of a lower SES were less likely to receive IRF care in North Carolina. In another study of Maryland hospital discharge data, Onukwugha and Mullins reported that Blacks were more likely to receive institutional care, relative to Whites, following stroke.^19^ Others have reported similar findings, ^10,23,45^ though some have reported that Blacks and/or Hispanics are more likely to be discharged home.^23,46^ In a recent study of Kaiser Permanente data from Northern California, Sandel and colleagues ^45^ reported that Blacks were more likely to receive IRF care following stroke. In a follow-up study of HH and outpatient use during the year following stroke, minorities were more likely to receive more intensive care. ^47^

While data on racial disparities are mixed, most studies are consistent in reporting that use of institutional care is greater in older individuals and females ^30,45,48-50^ and that older females and individuals with a lower SES are more likely to receive less intensive PARC. ^45,47,51^ Studies are also consistent in reporting geographic variation in PARC use and greater PARC use in areas with more PARC supply.^45,52-56^

The objective of this study was to use population-based, hospital discharge data to determine, more specifically than in past studies, the extent to which demographic and geographic disparities exist PARC use following stroke. Based on previous literature, we hypothesized that demographic and geographic disparities would remain after controlling for illness severity/comorbidities, hospital characteristics, and PARC supply.

This study extends previous research by using current data on both Medicare and non-Medicare patients and by examining disparities in the use of the different types of PARC. We also extend previous research by including additional important covariates that may explain variation in PARC use.

## METHODS

### Research Design

We conducted a cross-sectional analysis of two years (2005, 2006) of population-based, hospital discharge data from short-term, acute care hospitals in four demographically and geographically diverse states (AZ, FL, NJ, WI). Records on patients admitted with a stroke diagnosis were identified. These data were merged with hospital, ZIP code, and county-level data.

### Conceptual Model

**Figure 1:**
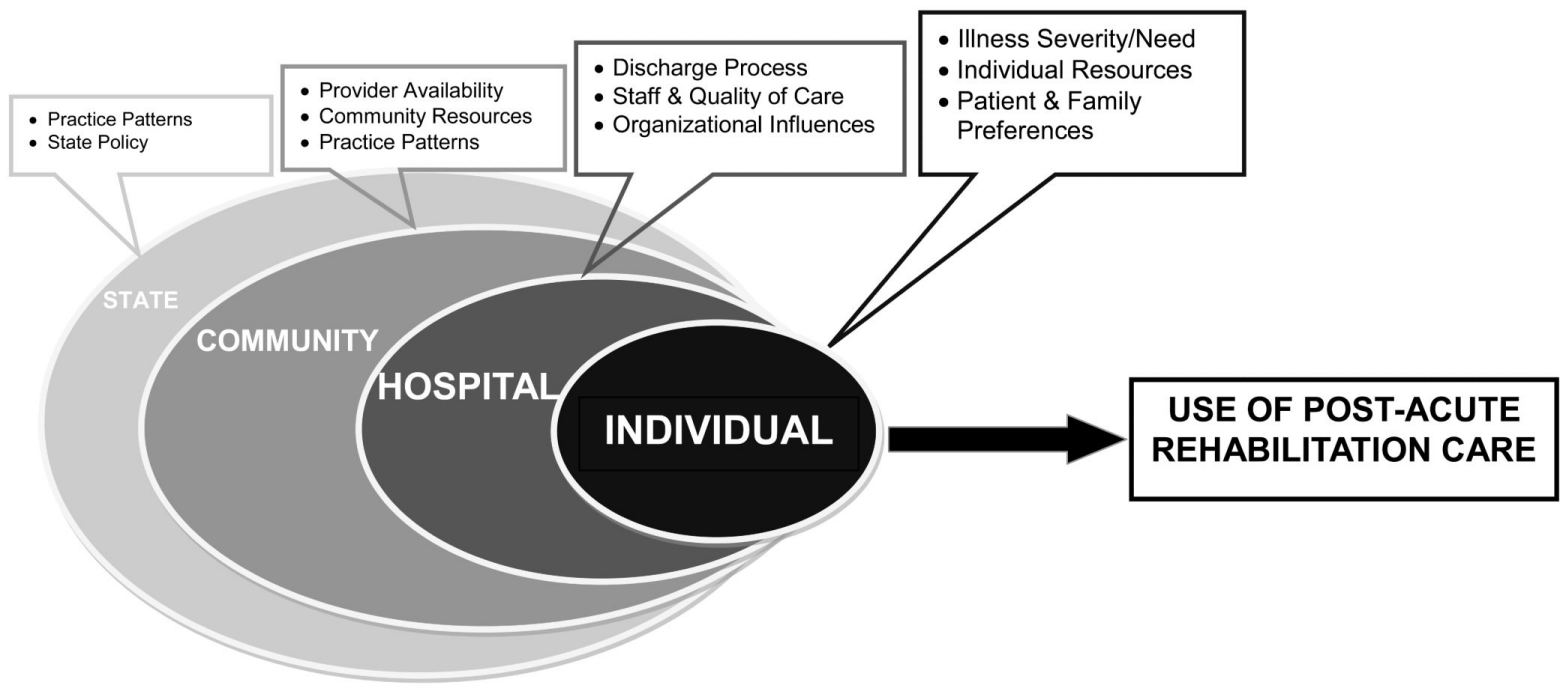
Conceptual Model

Based on the wealth of research on health care use, as well as the clinical experience of the research team, we hypothesized that factors at the individual, hospital, community, and state levels influence the type of PARC received following stroke (Figure 1). The figure also illustrates our hypothesis that factors more proximal to the patient have a stronger impact on PARC use.

### Data Sources

Our primary source of data was the State Inpatient Databases (SIDs).^57^ SIDs from AZ, FL, NJ, and WI were selected based on the availability of key data elements (e.g., race) and to get representation in the four U.S. census regions. Data on hospital characteristics were obtained from the American Hospital Association 2006 Annual Survey Database ^58^; the Centers for Medicare and Medicaid Services (CMS) 2006 Provider of Services File; ^59^ and the CMS 2006 Hospital Cost Reports.^60^ We used the 2006 Demographic Update of the Census 2000 data,^61^ created by Claritas, Inc. to obtain ZIP code-level data, and the 2006 Area Resource File ^62^ to obtain county-level data.

### Sample

**Figure 2:**
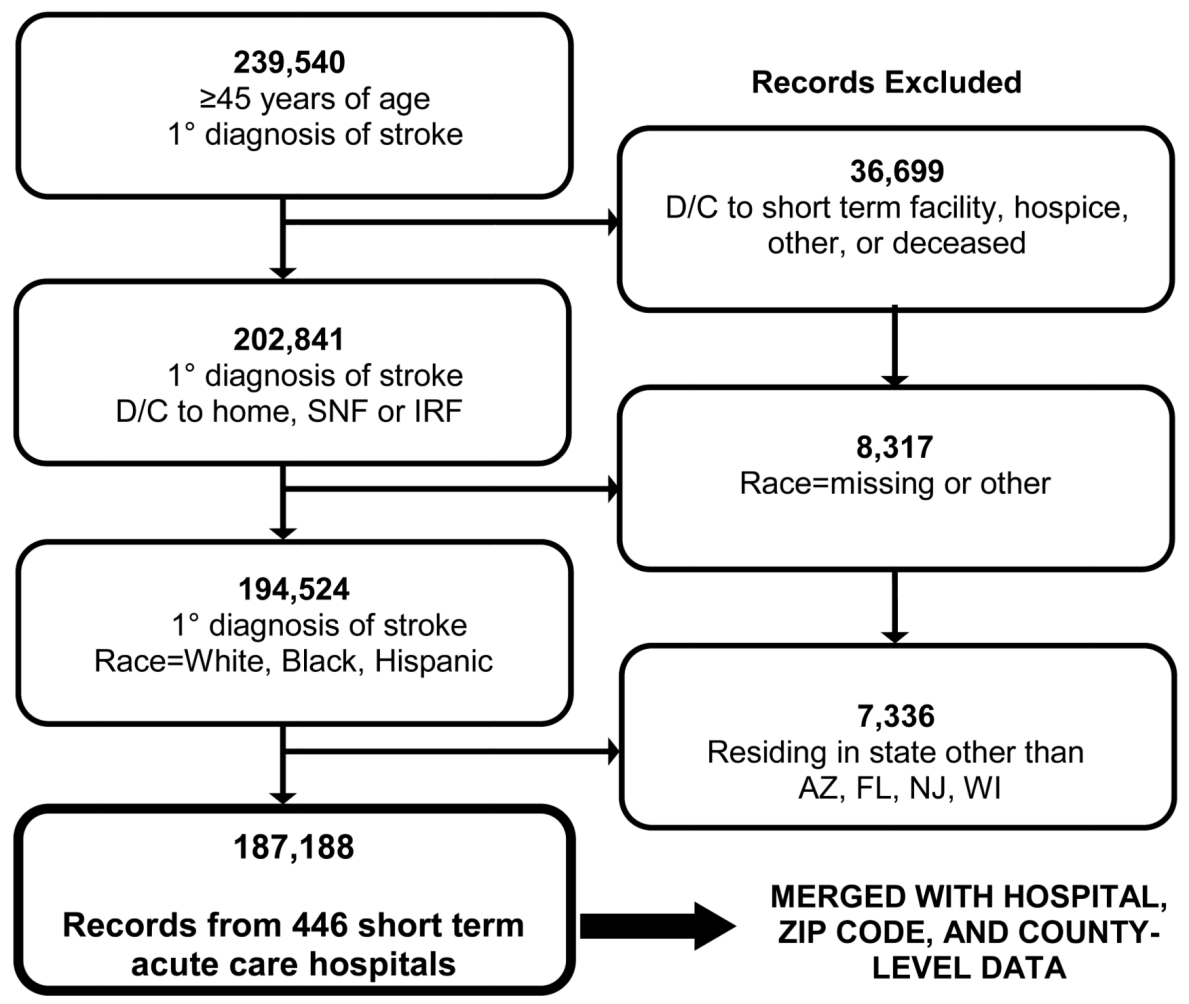
Creation of Final Sample

Our analysis was limited to individuals 45 years and older who were admitted to the hospital with a primary diagnosis of cerebrovascular disease (ICD-9-CM codes 430.×-438.×). We excluded individuals who died during their inpatient stay, were transferred to hospice care, were transferred to another short-term facility, were missing race data, or resided outside the study states (Figure 2). Due to small sample sizes, records with “other” race were excluded.

### Study Variables

The conceptual model and availability of data elements influenced our final variable selection. We created three dichotomous dependent variables: 1) whether the subject received institutional care; 2) for subjects who did not receive institutional care, whether they received home health care; and 3) for subjects who received institutional care, whether they received SNF or IRF care.

Our primary independent variables were race, sex, age, SES, metropolitan status of residence, and state. Race was coded White, Black, or Hispanic with ethnicity taking precedence over race. ^63^ “White” and “Black,” therefore, refer to non-Hispanic White and non-Hispanic Black. The subject’s SES was represented by two variables: insurance (being uninsured or on Medicaid) and median household income in the subject’s ZIP code.^64^ We used the National Center for Health Statistics urban/rural classification scheme and created a three-level categorization for the counties in which cases resided: large metropolitan; medium/small metropolitan; or micropolitan/non-micropolitan.^65^ We also controlled for the state in which the subject resided.

To account for clinical factors that may influence PARC use we included the following variables: length of stay (categorized as ≤2 days; 3-4 days; 5-6 days; and > 6 days); Emergency Department admission; stroke type (transient ischemic attack, hemorrhagic, ischemic/other);^66-68^ illness severity and mortality risk based on the All Patient Refined Diagnostic Related Groups scoring system;^69^ and comorbidity measures, derived from Elixhauser’s list of 29.^70^ We created indicators for select comorbidities based on the literature ^23,30,66,67,71,72^ and an indicator for individuals with 3 or more of the 29 comorbidities. We also included two indicators of vascular risk: atrial fibrillation (ICD-9-CM codes 427.×) and hypertensive heart disease (ICD-9-CM codes 402.×);^71^ an indicator for dementia (ICD-9-CM codes 290.× – 29.4.x, 331.×);^73^ and an indicator for the presence of dysphagia (ICD-9-CM codes 438.×). Descriptive data on the sample are presented in Table 1.

We included several hospital variables as proxies for quality of care. These included: stroke volume (i.e., number of stroke admissions averaged across the two years), ^23,74-76^ whether the hospital had a major medical school affiliation,^23,77-79^ registered nurse (RN) FTEs per 100 admissions,^80,81^ and physical, occupational, and speech therapists FTEs per 1000 admissions. The latter variable may be important because therapists’ time spent with the patient may impact the appropriateness of PARC. We also included a variable to indicate the for-profit status of the hospital because patient outcomes and incentives at for-profit differ from those at not-for-profit hospitals.^82-84^ Finally, to control for PARC supply,^53^ we included variables to indicate whether the hospital maintained a SNF, HH agency, or IRF; and county-level measures of the number of PTs/OTs, HH agencies, SNF beds, and IRFs, each standardized to the county population.

### Data Analysis

All analyses were conducted using SAS (v9.2) and a generalized linear mixed model approach with a logit link to account for the correlation of patients within hospital and to examine the associations between our independent variables and PARC use.(33) We estimated three separate, mixed-effects logistic regression models, one for each of our three dichotomous outcome variables. Because SIDs in Florida did not distinguish between the use of SNFs and IRFs in 2005, Florida data from this year were excluded from the SNF/IRF analysis.

For each outcome, the first level of our models included all independent variables at the patient, hospital, and county level (fixed effects). To account for unobserved heterogeneity in PARC use by hospital, we included hospital-specific random intercepts. To quantify the heterogeneity across hospitals, we calculated the median odds ratio (MOR).^85,86^ An MOR of 1 indicates no variation in PARC use due to unobserved factors between hospitals. The larger the MOR, the greater the variation in hospital intercepts.

We explored interactions between the level 1 race variables and socioeconomic (Medicaid and uninsured) and geographic (metropolitan and state) variables. Due to the limited number of significant interactions, these findings are presented in the text only.

## RESULTS

Our sample was 80 percent White and 52 percent female with a mean age of 73 years (Table 1). Over 90 percent of the sample had Medicare or private insurance and 63 percent lived in a large metropolitan area. Sixty-two percent of the sample suffered an ischemic stroke and 22 percent had a severity measure of major/extreme. The mean length of stay in acute care was 7.5 days.

Twenty-eight percent of the sample used institutional care. Of those discharged home, 17 percent received HH. Of those discharged to an institution, 69 percent received SNF care. Demographic, geographic, and clinical differences by PARC use were apparent in the descriptive analyses.

### Use of Institutional Care (Table 2)

In the multilevel analyses, Blacks, females, older individuals, those on Medicare or Medicaid, and those with lower incomes were more likely to receive institutional care. Subjects living in Wisconsin were most likely to be receive institutional care and those in Florida were least likely. Hispanic subjects and those without insurance were less likely to receive institutional care.

Few hospital or PARC supply characteristics were associated with institutional use. Subjects seen at hospitals that had a higher ratio of therapist FTE’s/admissions were more likely to receive institutional care, as were subjects living in counties with a higher ratio of HH agencies/county population.

The MOR was 1.52 indicating heterogeneity across hospitals in the propensity for individuals with similar characteristics to receive institutional care. The proportion of the unobserved variation in institutional use attributable to unobserved (i.e., unmeasured) hospital characteristics was 6 percent (.1944 / (.1944 + π^2^ / 3) = .056).

The relationship between race and institutional use was modified by insurance. Minorities on Medicaid or who were uninsured were less likely to receive institutional care relative to Whites who were uninsured or on Medicaid. Interactions between race and the geographic variables were not significant.

### Use of HH Care (Table 3)

Minorities, females, older individuals, those on Medicare, and those with lower incomes were more likely to receive HH. Uninsured individuals and those living in rural areas were less likely. Subjects living in Arizona and Wisconsin were least likely to receive HH and those in Florida were most likely.

Few hospital and PARC supply characteristics were associated with HH use. Subjects treated at hospitals with higher volumes of stroke admissions and those living in counties with a greater supply of PTs and OTs were more likely to receive HH.

The MOR was 1.80 indicating heterogeneity across hospitals in the propensity for individuals with similar characteristics to receive HH. The proportion of the unobserved variation in discharge status attributable to unobserved (i.e., unmeasured) hospital characteristics was 10 percent.

The relationship between race and use of HH was modified by state. Minorities in Wisconsin were much more likely to receive HH relative to Whites in Wisconsin. The other states had no evidence of within-state differences nor was there evidence of interactions between race and metropolitan status or insurance.

### Use of SNF versus IRF Care (Table 4)

Blacks; females; older individuals; those on Medicare, Medicaid, or uninsured; and those with lower incomes were more likely to use SNF care. Individuals who lived in metropolitan areas or in Florida were also more likely to use SNF care. NJ residents were more likely to receive IRF care.

Hospital characteristics associated with IRF use were higher stroke volume, for-profit status, and the presence of an affiliated IRF. None of the PARC supply variables were associated with SNF/IRF use. The MOR was 4.75, indicating considerable heterogeneity across hospitals in the propensity of an individual to use SNF versus IRF care. The proportion of unobserved variation in SNF versus IRF use attributable to unmeasured hospital characteristics was 45 percent.

As in the analysis of HH use, minorities in Wisconsin were more likely to use IRF care relative to Whites in Wisconsin. Interactions of race and the other geographic and insurance variables were not significant.

## DISCUSSION

We identified several demographic and geographic differences in PARC use after controlling for illness severity/comorbidities, hospital characteristics, and PARC supply. Considering PARC as a continuum of more to less intensive care (ie, IRF, SNF, HH, no HH) we found some consistent findings. Blacks, females, older individuals, and individuals living in communities with lower incomes were more likely to receive institutional care; and if discharged home, were more likely to receive HH. Considering the fact that, all other things equal, individuals with these characteristics tend to make smaller gains in recovery following stroke, ^4,11,16-19^ these findings are encouraging. While we controlled for several measures of illness severity/comorbidities, the association of more intensive care with these characteristics may be indicative of unmeasured illness severity.

Although some of our findings were positive, some were indicative of disparities in care. Uninsured and Hispanic individuals were less likely to receive institutional care and uninsured individuals and those living in rural areas were less likely to receive HH. We also found that Minorities on Medicaid or who were uninsured were less likely to receive institutional care relative to their Whites counterparts. In regard to SNF versus IRF use, we found that Blacks, females, older individuals, those with lower incomes, and the uninsured were less likely to use IRF care. While some of our findings regarding these associations may be explained by unmeasured illness severity, we believe these findings are also indicative of potential disparities in care. Studies suggest that patients seen at IRFs make greater functional gains following stroke than patients seen at SNFs.^87,88^

We found significant state to state variation in PARC use. The effect of race was also modified by state. Relative to the other explanatory variables, state had a large impact on PARC use, which was contrary to what we hypothesized, but consistent with a body of literature showing that where you live has a large influence on the quality of care you receive.^89,90^ What is notable is that the state effects are large even though our models included PARC supply variables and hospital-specific intercepts.

Our findings regarding the association of hospital characteristics on PARC use revealed some consistent findings regarding volume of stroke patients, a quality of care marker. ^23,74-76^ Individuals seen at hospitals with a higher stroke volume were more likely to receive HH care if discharged home and more likely to receive IRF care if discharged to an institution. While stroke volume was not associated use of institutional care, we did find that individuals seen in hospitals with a higher ratio of therapist FTE’s/admissions were more likely to receive institutional care.

The MORs for our models ranged from 1.52 – 4.75 and the unexplained variation due to unmeasured hospital characteristics ranged from 6 – 45 percent, indicating heterogeneity across hospitals in the propensity of individuals with *similar* characteristics to use the same type of PARC. These findings suggest that the hospital at which an individual is treated will have an impact on the type of PARC they receive, particularly in regard to SNF versus IRF care where the heterogeneity was greatest.

Only a few of the PARC supply variables were associated with PARC use, generally in the expected manner. Local availability of PARC is a major determinant of whether an individual receives that type of care. ^53^ One explanation for some of the non-significant findings in our analyses may be the lack of precision in our supply measures.

### Study Limitations

We examined data from only four states and found PARC use varied considerably by state. Our findings may not be generalizable to other states. We also did not have any direct measures of the patient’s functional status. We used multiple measures of illness severity/comorbidities as proxies for functional status. While almost all of these were significant in the expected directions, we likely did not account for all of the variation in functional status. We also did not have information on the use of outpatient therapy. It is unclear whether individuals who did not receive HH received outpatient therapy. Finally, our models lacked direct measures of patient preferences and provider characteristics.

Despite these limitations, this study adds to the limited, recent literature on disparities in PARC use for stroke. Documenting disparities can help identify areas to target, provide new hypotheses regarding determinants of disparities, and identify new avenues for the elimination of these disparities. Based on our findings, efforts at the state-level may be most effective. Although we explored various interaction effects, the interplay between individual and contextual factors needs further exploration and could be a critical determinant in PARC use.

## CONCLUSIONS

After controlling for illness severity/comorbidities, hospital characteristics, and community-level factors, demographic and geographic differences in PARC use following stroke remained. Some of these differences appear to be indicative of racial, socioeconomic, and geographic disparities in care. Because the burden of stroke is greater for minorities and individuals of lower SES, efforts to minimize these disparities and to further our understanding of the reasons behind them are needed.

## Figures and Tables

**Table 1:** Demographic, Geographic, & Clinical Characteristics of Sample (N=187,188)

		By Discharge Status^1^			
		Home (71.9%)		Institution (28.1%)	
		
	Entire Sample (100%)	Home (83.4%)	HH (16.6%)	IRF (31.1%)	SNF (68.9%)
**DEMOGRAPHIC & GEOGRAPHIC**					

**Female (%)**	52.4	48.6	57.9	51.3	61.2
**Race (%)**					
White	79.5	80.5	75.7	77.9	80.7
Black	11.4	9.9	13.9	13.5	12.9
Hispanic	9.1	9.6	10.4	8.6	6.5
**Mean(SD) Age, y**	72.6 (11.9)	69.7 (11.5)	75.7 (11.1)	72.5 (11.9)	79.4 (10.4)
**Insurance (%)**					
Private	20.2	26.3	12.1	73.0	87.0
Medicare	72.4	64.8	82.5	20.7	7.8
Medicaid	3.6	3.9	3.1	4.1	3.1
None/Self-Pay	3.7	5.1	2.2	2.3	1.1
**Median HH Income^3^ (%)**					
Highest quartile	22.1	21.9	19.4	31.9	25.6
Quartile 3	24.2	24.5	23.4	24.0	24.6
Quartile 2	26.8	27.0	28.0	21.6	26.4
Lowest quartile	26.9	26.6	29.3	22.5	23.4
**Patient residence^4^ (%)**					
Micropolitan, non-metro area	9.1*	9.7	5.9	7.5	10.7
Medium-small metro area	27.6*	28.2	26.4	28.2	24.1
Large metro area	63.3*	62.0	67.7	64.3	65.2
**Patient State (%)**					
Arizona	12.2	13.4	8.3	18.2	12.4
Florida	54.3	55.3	67.3	23.2^2^	36.8^2^
New Jersey	22.0	19.7	19.1	39.3	33.7
Wisconsin	11.6	11.6	5.3	19.4	17.2

**CLINICAL**					

**ED admission (%)**	67.7	60.5	74.5	79.5**	79.1**
**Mean (SD) length of stay, days**	7.5 (8.1)	3.1 (3.2)	5.1 (5.2)	7.4 (7.2)**	7.5 (8.3)**
**Type of Stroke (%)**					
Transient Ischemic Attack (TIA)	26.8	34.2	25.2	3.0	15.4
Ischemic	61.9	57.7	62.6	76.5	68.1
Hemorrhagic	2.2	1.5	2.7	3.2	3.3
Other	9.2	6.6	9.6	17.3	13.3
**APR-DRG severity measure (%)**					
Minor	25.7	36.1	16.0	7.8	7.6
Moderate	52.5	52.1	57.8	53.6	49.9
Major/extreme	21.9	11.8	26.2	38.6	42.5
**APR-DRG mortality risk (%)**					
Minor	45.0	60.3	31.9	26.2	15.1
Moderate	44.1	34.7	55.4	54.9	61.3
Major/extreme	10.8	5.0	12.7	18.9	23.7
**Comorbidities (%)**					
Renal Failure	6.4	4.9	8.2	7.4	10.5
Psychoses	2.1	1.6	2.3	1.7	3.5
Diabetes with complications	4.2	3.6	5.7	4.8**	5.2**
Depression	7.0	6.1	7.7	7.6	9.9
Congestive heart failure	7.4	4.1	8.9	11.4	14.8
Chronic pulmonary disease	15.8	15.1	17.6	14.6	17.1
3 or more comorbidities	33.7	27.9	40.0	40.5	45.4
Atrial fibrillation	16.5	11.2	21.6	22.7	27.0
Hypertensive heart disease	2.4	2.0	3.0	2.5**	2.9**
Dementia	5.2	2.3	6.5	3.5	13.3
Speech impairments/dysphagia	1.7	1.1	2.1	2.3	3.3

1 all comparisons significantly different (p<.01) unless indicated

2 only 2006 FL data are included, in 2005 the FL SID did not distinguish between IRF and SNF discharges

3 based on patient zip-code

4 based on patient county of residence

* no statistically significant difference in proportion discharged to institution vs home

** no statistically significant difference in proportion discharged to IRF vs SNF

**Table 2:** Mixed Effects Logistic Regression Analysis, Institution versus Home^1^ (N= 185,997)^2^

		Odds Ratio	P value	95% CI	
***Sociodemographic***					
Race:	White (referent)	1.00	---	---	---
	Black	1.34	<.0001	1.28	1.40
	Hispanic	0.83	<.0001	0.78	0.87
Sex:	Female	1.13	<.0001	1.10	1.16
Age:^3^	Age/10	1.57	<.0001	1.54	1.59
Insurance:	Private(referent)	1.00	---	---	---
	Medicare	1.31	<.0001	1.25	1.36
	Medicaid	1.41	<.0001	1.30	1.51
	Uninsured	0.49	<.0001	0.45	0.54
Median Income:^4^	Highest quartile (referent)	1.00	---	---	---
	Quartile 3	1.06	0.01	1.01	1.10
	Quartile 2	1.08	0.002	1.03	1.13
	Quartile 1	1.11	<.0001	1.05	1.16
***Geographic***					
Metro Status:	Micropolitan/rural (referent)	1.00	---	---	---
	Medium	1.00	0.95	0.92	1.09
	Large	0.99	0.89	0.91	1.09
State:	WI (referent)	1.00	---	---	---
	AZ	0.59	<.0001	0.49	0.72
	FL	0.41	<.0001	0.35	0.49
	NJ	0.56	<.0001	0.47	0.67
***Hospital Characteristics***					
Stroke volume/100		1.00	0.83	0.98	1.02
PT, OT, &ST FTEs/1000 admissions		1.04	0.002	1.01	1.06
RN FTEs/100 admissions		1.00	0.95	0.95	1.05
Major Medical School Affiliation		1.00	0.99	0.86	1.17
For Profit Hospital		0.98	0.77	0.86	1.12
***PARC Supply at Hospital***					
Has nursing home unit		1.14	0.11	0.97	1.34
Has an IRF		0.89	0.08	0.79	1.01
Has a HHA		1.14	0.02	1.02	1.27
***PARC Supply in County of Patient’s Residence***					
PTs & OTs./10,000		1.01	0.12	1.00	1.01
HHAs/100,000		1.06	<.0001	1.04	1.08
SNF beds./1,000 ≥65 yrs.		0.99	0.17	0.98	1.00
IRFs/100,000 ≥65 yrs.		0.99	0.02	0.97	1.00

Random Effects Variance	Estimate	SE	P value	Median OR
Hospital	0.1944	0.1780	<.0001	1.52

1 home is base category & controlling for disease severity, comorbidities, length of stay, and clinical variables;

2 records with missing observations dropped

3 age divided by 10 to assist in interpretation of odds ratio

4 median household income for the zip code of patient’s residence

**Table 3:** Mixed Effects Logistic Regression Analysis, HH versus Home with No HH^1^ (N=133,622)^2^

		Odds Ratio	P value	95% CI	
***Sociodemographic***					
Race:	White (referent)	1.00	---	---	---
	Black	1.56	<.0001	1.47	1.65
	Hispanic	1.14	<.0001	1.07	1.21
Sex:	Female	1.33	<.0001	1.29	1.38
Age:^3^	Age/10	1.51	<.0001	1.48	1.54
Insurance:	Private(referent)	1.00	---	---	---
	Medicare	1.41	<.0001	1.34	1.49
	Medicaid	1.09	0.09	0.99	1.20
	Uninsured	0.60	<.0001	0.53	0.66
Median Income:^4^	Highest quartile (referent)	1.00	---	---	---
	Quartile 3	1.03	0.24	0.98	1.09
	Quartile 2	1.09	0.003	1.03	1.16
	Quartile 1	1.10	0.002	1.04	1.17
***Geographic***					
Metro Status:	Micropolitan/rural (referent)	1.00	---	---	---
	Medium	1.24	0.000	1.10	1.40
	Large	1.34	<.0001	1.18	1.51
State:	WI (referent)	1.00	---	---	---
	AZ	1.10	0.51	0.83	1.47
	FL	2.14	<.0001	1.68	2.71
	NJ	1.42	0.006	1.11	1.82
***Hospital Characteristics***					
	Stroke volume/100	1.05	0.003	1.02	1.08
	PT, OT, &ST FTEs/1000 admissions	0.97	0.50	0.91	1.05
	RN FTEs/100 admissions	1.01	0.45	0.98	1.05
	Major Medical School Affiliation	0.85	0.14	0.68	1.06
	For Profit Hospital	1.10	0.29	0.92	1.32
***PARC Supply at Hospital***					
	Has nursing home unit	0.83	0.14	0.65	1.06
	Has an IRF	0.99	0.87	0.82	1.18
	Has a HHA	1.16	0.06	0.99	1.36
***PARC Supply in County of Patient’s Residence***					
	PTs & OTs./10,000	1.02	0.006	1.00	1.03
	HHAs/100,000	0.99	0.22	0.97	1.01
	SNF beds./1,000 ≥65 yrs.	1.04	0.01	1.01	1.06
	IRFs/100,000 ≥65 yrs.	1.00	0.72	0.98	1.02

Random Effects Variance	Estimate	SE	P value	Medicin OR
Hospital	0.3784	0.0363	<.0001	1.80

1 home is base category & controlling for disease severity, comorbidities, length of stay, and clinical variables

2 records with missing observations dropped

3 age divided by 10 to assist in interpretation of odds ratio

4 median household income for the zip code of patient’s residence

**Table 4:** Mixed Effects Logistic Regression Analysis, SNF versus IRF^1^ (N=41,386)^2^

		Odds Ratio	P value	95% CI	
***Sociodemographic***					
Race:	White (referent)	1.00	---	---	---
	Black	1.21	<.0001	1.10	1.32
	Hispanic	0.98	0.68	0.87	1.09
Sex:	Female	1.26	<.0001	1.19	1.33
Age:^3^	Age/10	1.73	<.0001	1.67	1.78
Insurance:	Private(referent)	1.00	---	---	---
	Medicare	1.21	<.0001	1.11	1.32
	Uninsured	2.84	<.0001	2.43	3.31
	Medicaid	1.56	<.0001	1.26	1.94
Median Income:^4^	Highest quartile (referent)	1.00	---	---	---
	Quartile 3	1.16	0.001	1.06	1.16
	Quartile 2	1.26	<.0001	1.15	1.26
	Quartile 1	1.28	<.0001	1.16	1.28
***Geographic***					
Metro Status:	Micropolitan/rural (referent)	1.00	---	---	---
	Medium	1.18	0.06	0.99	1.41
	Large	1.32	0.006	1.08	1.60
State:	WI (referent)	1.00	---	---	---
	AZ	0.79	0.51	0.40	1.58
	FL	2.38	0.005	1.29	4.39
	NJ	0.37	0.002	0.20	0.69
***Hospital Characteristics***					
	Stroke volume/100	0.84	0.001	0.76	0.93
	PT, OT & ST FTEs/1000 admissions	1.03	0.51	0.94	1.13
	RN FTEs/100 admissions	0.98	0.88	0.81	1.20
	Major Medical School Affiliation	0.53	0.03	0.30	0.94
	For Profit Hospital	0.42	0.001	0.25	0.70
***PARC Supply in Hospital***					
	Has Nursing home unit	1.72	0.08	0.93	3.17
	Has an IRF	0.23	<.0001	0.14	0.35
	Has a HHA	1.51	0.05	0.99	2.29
***PARC Supply in County of Patient’s Residence***					
	PTs & OTs./10,000	1.00	0.54	0.98	1.01
	HHAs/100,000	1.00	0.96	0.97	1.03
	SNF beds./1,000 ≥65 yrs.	1.02	0.34	0.98	1.06
	IRFs/100,000 ≥65 yrs.	0.98	0.02	0.95	1.00

Random Effects Variance	Estimate	SE	P value	Median OR
Hospital	2.6715	0.2436	<.0001	4.75

1 IRF is base category & controlling for disease severity, comorbidities, length of stay, and clinical variables

2 Florida 2005 data excluded from analysis as well as records with missing data

3 age divided by 10 to assist with interpretation of odds ratio

4 median household income for zip code of patient’s residence
